# *Salmonella typhimurium* exploits macrophage-derived arginine as an intracellular signal for SPI-2 virulence gene activation

**DOI:** 10.3389/fcimb.2026.1877832

**Published:** 2026-09-21

**Authors:** Xinyue Wang, Shuai Ma, Ting Ye, Houliang Guo, Yiming Wang, Xiaolin Yan, Yuyang Sun, Mengjie Zhao, Lingyan Jiang, Lu Feng

**Affiliations:** 1 National Key Laboratory of Intelligent Tracking and Forecasting for Infectious Diseases, TEDA Institute of Biological Sciences and Biotechnology, Nankai University, Tianjin, China; 2 School of Synthetic Biology and Biomanufacturing, Tianjin University, Tianjin, China

**Keywords:** arginine, ArgR, *Salmonella typhimurium*, SPI-2 T3SS, virulence regulation

## Abstract

**Background:**

Arginine contributes to host defense by serving as a substrate for inducible nitric oxide synthase (iNOS) to produce antimicrobial nitric oxide (NO). However, how bacterial pathogens respond to host arginine metabolism remains poorly understood.

**Results:**

We found that *Salmonella* Typhimurium (*S*. Tm) infection causes arginine accumulation in macrophages. This accumulation is associated with two concurrent mechanisms: enhanced arginine import via upregulation of the CAT2 transporter, and pathogen-mediated suppression of host iNOS expression. *S*. Tm then exploits this accumulated arginine as an intracellular signal to upregulate virulence gene expression and promote its replication. Mechanistically, arginine activates the type III secretion system (T3SS) encoded by *Salmonella* pathogenicity island 2 (SPI-2) via the bacterial transcriptional regulator ArgR, which binds to the promoter of *ssrA*, the key regulator of SPI-2, in an arginine-dependent manner. Disruption of *argR* impairs *S*. Tm intracellular replication and attenuates virulence in mice.

**Conclusions:**

These findings reveal that *S*. Tm co-opts the host’s arginine-mediated immune response by sensing macrophage-derived arginine as a signal to initiate its virulence program, revealing a pathogen adaptation strategy that links metabolic sensing to virulence activation.

## 1 Introduction

*Salmonella enterica* serovar Typhimurium (*S.* Tm) is a Gram-negative facultative intracellular pathogen responsible for a spectrum of diseases in humans and animals, ranging from self-limiting gastroenteritis to severe systemic infections that can be fatal (Coburn et al., 2007; Fàbrega and Vila, 2013; Galán-Relaño et al., 2023). Following oral ingestion, *S.* Tm traverses the gastric barrier, invades the intestinal epithelium, and subsequently disseminates to systemic sites such as the mesenteric lymph nodes, liver, and spleen (Watson and Holden, 2010; Li et al., 2023). A critical determinant for the establishment of systemic infection by *S.* Tm is its ability to survive and replicate within innate immune cells, particularly macrophages (Fields et al., 1986; Leung and Finlay, 1991). Within these cells, *S.* Tm resides in a specialized membrane-bound compartment known as the *Salmonella*-containing vacuole (SCV) (Steele-Mortimer, 2008). The SCV not only protects the pathogen from host microbicidal mechanisms but also serves as a replicative niche by facilitating nutrient acquisition from the host (Knuff and Finlay, 2017; Liss et al., 2017). The biogenesis and maintenance of the SCV depends on an array of virulence determinants, most notably the type III secretion system (T3SS) encoded by *Salmonella* pathogenicity island 2 (SPI-2) (Figueira and Holden, 2012; Jennings et al., 2017). Expression of the SPI-2 T3SS is controlled by a complex regulatory network and is induced following phagocytosis in response to specific intracellular cues, such as low pH, limited Mg^2+^, phosphate restriction, and Fe^2+^ scarcity (Deiwick et al., 1999; Löber et al., 2006; Jiang et al., 2020).

Macrophages play a central role in innate immunity and serve as a first line of defense against invading pathogens (Chen et al., 2023). Upon recognition of conserved microbial features known as pathogen-associated molecular patterns (PAMPs), such as lipopolysaccharide (LPS), macrophages undergo activation toward a pro-inflammatory phenotype (Cerdeira and Brigagão, 2024). This activation triggers a robust antimicrobial response, involving the production of reactive oxygen/nitrogen species (ROS/RNS) and the secretion of pro-inflammatory cytokines (Orecchioni et al., 2019). Supporting these energy-intensive immune responses requires profound metabolic reprogramming, encompassing extensive remodeling of the host metabolic network (Galván-Peña and O'Neill, 2014). However, intracellular pathogens including *S*. Tm have evolved strategies to adapt to or exploit these metabolic alterations, thereby circumventing host restrictions on bacterial replication (Rosenberg et al., 2022). The shift in glucose metabolism, characterized by upregulation of glycolysis coupled with suppression of mitochondrial oxidative phosphorylation (OXPHOS) and reduced tricarboxylic acid (TCA) cycle activity is the key feature in host metabolic reprogramming (Escoll and Buchrieser, 2018). *S*. Tm has developed mechanisms to interfere with this host metabolic shift, utilizing metabolic intermediates either as nutrients—for example, 3-phosphoglycerate—to fuel its intracellular replication (Jiang et al., 2021), or as signaling molecules—such as succinate and lactate—to induce virulence gene expression (Rosenberg et al., 2021; Wang et al., 2023). Beyond glucose metabolism, alterations in other metabolic pathways, including arginine metabolism, have also been reported during infection (Das et al., 2010; Ma et al., 2025). However, how *S*. Tm adapts to these broader metabolic changes remains poorly characterized. Importantly, emerging evidence indicates that metabolic reprogramming is not restricted to macrophages; remodeling of other immune compartments also significantly influences host resistance to *S.* Tm infection. For instance, dietary conjugated linoleic acid has been shown to enhance resistance to *S.* Tm by promoting PPARγ-mediated metabolic reprogramming and effector function in CD8^+^ T cells (Deng et al., 2026). This finding broadens the landscape of host-pathogen metabolic interactions, highlighting that immunometabolic modulation extends beyond the macrophage compartment to include adaptive immune cells.

Arginine, as the exclusive substrate for the synthesis of the antimicrobial effector molecule nitric oxide (NO) and subsequent reactive nitrogen species (RNS), plays a pivotal role in the immune responses of macrophages (Mori and Gotoh, 2000; Popovic et al., 2007; Morris, 2010; Martí and Reith, 2021; Fung et al., 2025). The metabolism of arginine is primarily mediated by two key enzymes, arginase and inducible nitric oxide synthase (iNOS) (Rath et al., 2014). Under homeostatic conditions (non-infection), arginine is predominantly catabolized through arginase to produce ornithine and urea (Heuser et al., 2025). Upon pathogen infection, immune activation leads to the pronounced upregulation of iNOS, redirecting arginine flux toward the production of NO and citrulline (Cinelli et al., 2020). This metabolic reprogramming leads to a dramatic increase in arginine consumption that exceeds the capacity of endogenous synthesis, rendering macrophages highly dependent on extracellular arginine pools (Morris, 2016). To meet this elevated demand for NO generation, macrophages upregulate the expression of cationic amino acid transporter 2 (CAT2/Slc7a2), thereby enhancing arginine uptake (Deves and Boyd, 1998; Yeramian et al., 2006). Consequently, the rewiring of arginine metabolism sustains robust RNS production, creating a potent antimicrobial environment that effectively restricts the survival and replication of intracellular pathogens (Fang, 2004).

Multiple bacterial pathogens have evolved strategies to manipulate host arginine metabolism, subverting RNS-mediated immunity to promote their survival. For example, *Mycobacterium tuberculosis* promotes the expression of arginase in mouse macrophages, resulting in decreased intracellular NO production and a weakened antibacterial response from the host (El Kasmi et al., 2008). *Helicobacter pylori* expresses an arginase that competes with host iNOS for arginine, thereby reducing intracellular NO production (Gobert et al., 2001). In the case of *S.* Tm, previous studies have shown that the pathogen employs the small RNA fragment Sal-1 to inhibit host iNOS induction, dampening NO production and enhancing bacterial survival (Gu et al., 2017; Zhao et al., 2017). However, it remains unclear how iNOS repression globally reshapes host arginine-related metabolites during *S.* Tm infection, and how the pathogen adapts to these metabolic changes to optimize its fitness and virulence.

In this study, we employed an integrative approach combining metabolomic profiling, *in vitro* cellular infection models, mouse experiments, and various molecular biology techniques to systematically investigate metabolic alterations in *S.* Tm-infected macrophages and the corresponding bacterial adaptive mechanisms. Our results reveal that *S.* Tm infection increased arginine availability in macrophages, driven by the concurrent effects of enhanced arginine import and pathogen-mediated suppression of iNOS-dependent consumption. Furthermore, we demonstrate that *S*. Tm senses intracellular arginine through the transcriptional regulator ArgR, which subsequently activates expression of the SPI-2 T3SS, thereby enhancing intracellular replication and systemic infection. In summary, our findings uncover a metabolic adaptation strategy in which *S*. Tm exploits host-derived arginine as a signal to enhance its pathogenicity.

## 2 Materials and methods

### 2.1 Bacterial strains, plasmid and growth conditions

The bacterial strains and plasmids used in this study are listed in **Supplementary Table 1**. The primer sequences used for the generation and confirmation of strains and plasmids are provided in **Supplementary Table 2**. The *Salmonella enterica s*erovar Typhimurium (*S.* Tm) strain ATCC 14028s used as the wild-type (WT) strain throughout this study. Mutant strains, including Δ*argR*, Δ*ssrB*, Δ*ssrB*Δ*argR*, and the FLAG-tagged strain *sifA*-FLAG were generated using the λ-Red recombinase system (Murphy and Campellone, 2003). The complemented strain cArgR was constructed by replacing the *phoN* locus in the Δ*argR* mutant with the native *argR* gene, allowing for *in situ* chromosomal complementation. For ArgR protein expression, the *argR* DNA fragment was cloned into the HindIII and BamHI sites downstream of the His‐tag element in plasmid pET-28a(+), resulting in the recombinant plasmid pET28a-*argR*. This plasmid was then transformed into *E. coli* BL21(DE3) for protein expression. Transcriptional fusion plasmids (*ssrA*-*lux, ssrB*-*lux*, and *sifA*-*lux*) were generated by PCR-amplifying the respective promoter regions from WT genomic DNA, digesting the products with BamHI and XhoI, and ligating them into the BamHI–XhoI site upstream of the *lux* genes in the vector pMS402, which carries a promoterless *luxCDABE* reporter cluster. The resulting reporter plasmids were transformed into the WT strain to generate the corresponding lux reporter strains: WT+*ssrA*-*lux*, WT+*ssrB*-*lux*, and WT+*sifA*-*lux*.

Bacterial strains were typically grown at 37 °C in Luria-Bertani (LB) medium (10 g/L tryptone, 5 g/L yeast extract, and 10 g/L NaCl) while being shaken at 180 rpm. Alternatively, they were grown on LB agar plates. When required, antibiotics were added at the following final concentrations: chloramphenicol (Cm), 25 μg/mL; kanamycin (Km), 50 μg/mL; ampicillin (Ap), 100 μg/mL; and gentamicin (Gm), 10 or 100 μg/mL. For qRT-PCR analysis, bacteria were initially grown overnight in LB medium at 37 °C, then subcultured at a 1:100 ratio into N-minimal medium (10 mM Tris–HCl [pH 7.5], 10 μM MgCl_2_, 1 mM KH_2_PO_4_, 0.5 mM K_2_SO_4_, 5 mM KCl, 7.5 mM (NH_4_)_2_SO_4_, 38 mM glycerol, and 0.1% [w/v] Casamino acids). Where indicated, the N-minimal medium was further supplemented with 0.1 mM or 1.2 mM arginine. Bacteria were incubated at 37 °C with shaking at 180 rpm for 6 h before being harvested by centrifugation for RNA extraction.

### 2.2 Macrophage culture

The murine RAW264.7 macrophage cell line (ATCC, Cat# TIB-71, RRID: CVCL_0493) was obtained from the Committee on Type Culture Collection of the Chinese Academy of Sciences (CTCC; Shanghai, China). Cells were maintained in RPMI-1640 medium supplemented with 10% (v/v) heat-inactivated fetal bovine serum (FBS) at 37 °C under a 5% CO_2_ atmosphere.

Bone marrow-derived macrophages (BMDMs) were isolated from female BALB/c mice by flushing the femurs and tibiae with RPMI-1640 medium. The collected bone marrow cells were resuspended in RPMI-1640 medium containing 10% FBS and 10 ng/mL recombinant murine macrophage colony-stimulating factor (M-CSF) at a density of approximately 1 × 10^6^ cells/mL. The cells were then seeded into 10-cm tissue culture plates at a density of approximately 5 × 10^6^ cells per plate and cultured at 37 °C in 5% CO_2_. The medium was refreshed on days 3 and 6 of culture. By day 7, the BMDMs were fully differentiated and suitable for subsequent infection experiments.

### 2.3 Macrophage infection

Bacterial strains were grown overnight at 37 °C with shaking in LB medium to stationary phase (OD_600_ ~ 2.0). The cultures were then serially diluted to 1 × 10^6^ CFU/mL in RPMI-1640 medium supplemented with 10% FBS and opsonized for 20 min. The opsonized bacteria were added to BMDMs or RAW264.7 cell monolayers (seeded at 1×10^5^ cells per well) at a multiplicity of infection (MOI) of 10. Infection was synchronized by centrifugation at 500 × g for 5 min. Following a 45-min incubation at 37 °C in a 5% CO_2_ atmosphere, cells were washed three times with PBS to remove non-internalized bacteria and then incubated in fresh RPMI-1640 medium containing 100 μg/mL gentamicin for 2 h to kill extracellular bacteria. The medium was subsequently replaced with RPMI-1640 containing 10 μg/mL gentamicin and maintained for the remainder of the experiment. For arginine modulation experiments, macrophages were cultured in RPMI-1640 supplemented with either 1.2 mM (standard formulation, ~1.14 mM) or 0.1 mM L-arginine, the latter chosen to represent a physiologically arginine-limited condition (Martí and Reith, 2021). Where indicated, 100 μM nor-NOHA (Nω-Hydroxy-nor-L-arginine) and 100 μM L-NMMA (N^G^-monomethyl-L-arginine, Tilarginine) were added to the cells along with the 10 μg/mL gentamicin. At specified time points post-infection, macrophages were lysed with 1% Triton X-100. Intracellular bacteria were harvested for quantification either by plating on LB agar for CFU enumeration or by processing for qRT-PCR analysis. The fold change in intracellular replication was calculated as the ratio of CFUs at 20 h post-infection (hpi) to CFUs at 2 hpi.

### 2.4 Immunofluorescence microscopy

To quantify intracellular bacteria, RAW264.7 cells were seeded on 20-mm-diameter coverslips and infected with *S.* Tm WT-GFP using the infection protocol described above. At 2 and 20 hpi cells were washed three times with PBS, fixed with 4% paraformaldehyde (PFA) at 4 °C for 15 min, and stained with DAPI at room temperature for 10 min. Coverslips were mounted and imaged using a confocal laser scanning microscope (Zeiss LSM800, Germany). Image processing and analysis were performed using ZEN 2.3 software (blue edition).

### 2.5 Metabolomics analysis

BMDMs were seeded in 10-cm tissue culture plates and either remained uninfected or were infected with *S.* Tm wild-type at a MOI of 10 for 8 h. After infection, the culture medium was aspirated, and cells were washed gently with PBS. Metabolites were extracted by adding 1 mL of cold methanol/acetonitrile/dH_2_O (40:40:20, v/v/v) to precipitate proteins. The resulting mixture was transferred to a new centrifuge tube and centrifuged at 14,000 × *g* for 20 min at 4 °C. The supernatant was collected and dried under vacuum centrifugation. Dried metabolites were reconstituted in 100 µL of acetonitrile/water (1:1, v/v), followed by centrifugation at 14,000 × *g* for 15 min to remove insoluble debris.

Metabolite profiling was performed using an ultra-high-performance liquid chromatography (UHPLC) system (1290 Infinity LC, Agilent Technologies) coupled to a quadrupole time-of-flight mass spectrometer (TripleTOF 6600, AB Sciex) at Shanghai Applied Protein Technology Co., Ltd. Hydrophilic interaction liquid chromatography (HILIC) separation was carried out on an ACQUITY UPLC BEH column (2.1 mm × 100 mm, 1.7 µm; Waters, Ireland) maintained at 25 °C. The mobile phase consisted of solvent A (25 mM ammonium acetate and 25 mM ammonium hydroxide in water) and solvent B (acetonitrile), delivered at a flow rate of 0.5 mL/min. Data were acquired in both electrospray ionization (ESI) positive and negative modes.

For untargeted metabolomics, six biological replicates (*n* = 6) were analyzed per condition, each derived from BMDMs isolated from an individual mouse. Raw mass spectrometry data (.wiff files) were converted to mzXML format using ProteoWizard MSConvert and processed with the XCMS platform for feature detection, alignment, and integration. Metabolites were identified by matching accurate mass (mass error < 10 ppm) and MS/MS fragmentation spectra against a custom in-house database established with authentic standards. After sum-normalization, multivariate statistical analyses, including Pareto-scaled principal component analysis (PCA) and orthogonal partial least squares-discriminant analysis (OPLS-DA), were performed using the R package ropls. For targeted metabolomics, four biological replicates (*n* = 4) were analyzed per condition, with all replicates representing independent macrophage cultures and infections. Metabolite identification was conducted through high-resolution mass and retention time matching against authentic standards. Significant differences between groups were assessed by Student’s *t* test. Metabolites with a variable importance in projection (VIP) > 1 and a *P*-value < 0.05 were considered statistically significant.

### 2.6 Mice

Female BALB/c mice (Cat# 213) aged six to eight weeks were obtained from Beijing Vital River Laboratory Animal Technology Co., Ltd. (Beijing, China). All the mice were kept in specific pathogen - free (SPF) conditions. A 12-h light/dark cycle was implemented, with the ambient temperature set at 24 ± 2 °C and the relative humidity at 50% ± 5%. Food and water were offered ad libitum.

### 2.7 *In-vivo* infection in mouse model

Mice were intraperitoneally (i.p.) inoculated with approximately 10^4^ colony-forming units (CFUs) of *S.* Tm wild-type, specified mutant strains, or complemented strains suspended in physiological saline. The survival status of infected mice was tracked and documented on a daily basis over a period of 16 days. To assess bacterial load in systemic organs (liver and spleen), mice were sacrificed by cervical dislocation on day 4 post-infection. Livers and spleens were collected under aseptic conditions, homogenized using ice-cold phosphate-buffered saline (PBS), serially diluted, and plated onto LB agar plates containing the appropriate antibiotics for CFU enumeration (CFUs per gram of tissue). For competitive infection assays, mice were co-inoculated i.p. with a 1:1 mixture of two strains. The competitive index (CI) was calculated using the following formula: CI = (CFU of strain A recovered/CFU of strain B recovered)/(CFU of strain A inoculated/CFU of strain B inoculated).

### 2.8 Fluorescence *in situ* hybridization (FISH)

Fluorescence *in situ* hybridization (FISH) was performed to detect CAT2 mRNA expression in liver and spleen cells infected with GFP-expressing wild-type *S.* Tm (*S*. Tm WT-GFP). FISH probes were designed and synthesized by Beijing Abace Biotechnology Co., Ltd. (Beijing, China). Infected mice were euthanized on day 4 post-infection, and liver and spleen samples were aseptically collected and embedded in OCT compound. Tissue sections (10 μm thickness) were prepared and fixed in 4% paraformaldehyde for 15 min, followed by washing with PBS. Hybridization was performed overnight at 37 °C using 10 nM probes in a buffer containing 40% formamide, 10% dextran sulfate, and 2 × SSC. Subsequently, sections were washed with 40% formamide in 1× SSC for 30 min at 37 °C, and then twice with 1× SSC for 10 min each at room temperature. Images were acquired using a confocal laser scanning microscope (Zeiss LSM800, Germany) and analyzed with ZEN 2.3 software (blue edition).

### 2.9 Small RNA interference

For siRNA-mediated knockdown of CAT2, RAW264.7 cells were transfected with either CAT2-specific siRNA (siRNA-CAT2) or negative control siRNA (siRNA-NC) using the riboFECT CP Transfection Kit (RIBOBIO, #C10511-05), 24 h prior to infection assays. All siRNA constructs were synthesized by Guangzhou RiboBio Biotechnology Co., Ltd. (Guangzhou, China). The sense strand sequence of the CAT2 siRNA was 5′–GTGGCTAACTGGAAGATCA–3′. Knockdown efficiency was assessed by quantifying CAT2 mRNA levels via qRT-PCR 24 hpi.

### 2.10 Sal-1 inhibition

To suppress Sal-1, a bacterial small RNA that represses host iNOS expression (Zhao et al., 2017), BMDMs were transfected with either a Sal-1 inhibitor or a negative control oligonucleotide (NC) using the riboFECT CP Transfection Kit (RIBOBIO, #C10511-05) 24 h before infection. The Sal-1 inhibitor (5′ –AATCCGTATCTTCGAGTGCCCACA – 3′) and NC oligonucleotide (5′ – UCUACUCUUUCUAGGAGGUUGUGA – 3′) were synthesized by Guangzhou RiboBio Biotechnology Co., Ltd. (Guangzhou, China). The efficiency of Sal-1 inhibition was verified by western blot analysis of iNOS protein levels at 8 hpi.

### 2.11 Quantitative real-time PCR (qRT-PCR)

Total RNA was isolated using TRIzol reagent and purified with an RNAsimple Total RNA Kit (TIANGEN #DP419) according to the manufacturer’s protocols. RNA concentration and purity were determined using a NanoDrop 2000 spectrophotometer (NanoDrop Technologies, USA). Complementary DNA (cDNA) was synthesized from total RNA using the StarScript II First-strand cDNA Synthesis Kit (Genstar, #A214). Quantitative PCR was performed using 2 × RealStar Green Fast Mixture (Genstar, #A301) with gene-specific forward and reverse primers in a total reaction volume of 20 µL. Amplification was carried out on a QuantStudio 5 Real-Time PCR System (Applied Biosystems, USA). The expression levels of *S*. Tm transcripts were normalized to the 16S rRNA gene, while those of macrophage or tissue origin were normalized to the *gapdh* gene. Relative fold changes in gene expression were computed using the 2^−ΔΔCt^ method (Livak and Schmittgen, 2001). All experiments were performed with three biological replicates, each including three technical replicates.

### 2.12 Bioluminescent reporter assays

To assess the effect of arginine on bacterial SPI-2 gene expression, overnight cultures of *S*. Tm wild-type strains harboring *ssrA*-lux, *ssrB*-lux, or *sifA*-lux transcriptional fusions were subcultured at a 1:1000 dilution into RPMI-1640 medium, either supplemented with 1.2 mM or 0.1 mM arginine, in a 96-well black-walled clear-bottom assay plate. The plates were incubated at 37 °C with continuous shaking. Luminescence and optical density at 600 nm (OD_600_) were measured at 30-min intervals using a Spark multimode microplate reader (Tecan, Switzerland). Relative promoter activity was expressed as luminescence units normalized to the OD_600_ value.

### 2.13 Western blotting

Protein lysates were prepared from cultured BMDMs or *S.* Tm strains using radioimmunoprecipitation assay (RIPA) buffer. Cells were lysed on ice for 30 min, followed by centrifugation at 12,000 rpm (4 °C) for 10 min. The supernatant was collected, mixed with 5 × SDS-PAGE loading buffer, and denatured at 99 °C for 10 min. Proteins were separated by SDS–PAGE on a SurePAGE™ gel (GenScript #M00658) and subsequently transferred to polyvinylidene difluoride (PVDF) membranes using a wet transfer apparatus. Membranes were blocked with 5% skim milk in Tris-buffered saline containing 0.1% Tween 20 (TBST) for 2 h at room temperature, then incubated overnight at 4 °C with the appropriate primary antibodies. The following primary antibodies were used: anti-Dnak antibody (1:5,000 dilution, Abcam Cat# ab80161, RRID: AB_1640416), anti-DDDDK tag (Binds to FLAG^®^ tag sequence) antibody (1:1,000 dilution, Abcam Cat# ab205606, RRID: AB_2916341), anti-iNOS antibody (1:1,000 dilution, Cell Signaling Technology Cat# 13120, RRID: AB_2687529) and anti-GAPDH antibody (1:1,000 dilution, (Cell Signaling Technology Cat# 5174, RRID: AB_10622025)). After washing, membranes were incubated with an HRP-conjugated goat anti-rabbit IgG secondary antibody (1:5,000 dilution, Sparkjade #EF0002) for 1 h at room temperature. Protein bands were visualized using the BeyoECL Star Ultra-sensitive ECL chemiluminescence kit (Beyotime #P0018AS) and imaged with an Amersham™ Imager 600 System (General Electric). Band intensities were quantified using ImageJ software.

### 2.14 Electrophoretic mobility shift assay (EMSA)

Recombinant His-tagged ArgR protein was purified by Ni-NTA affinity chromatography and validated via SDS-PAGE. The promoter region of the *ssrA* gene was amplified from genomic DNA of *S.* Tm wild-type strain, and the resulting PCR products were purified using the SanPrep Column PCR Product Purification Kit (Sangon Biotech, #B518141). For binding reactions, 50 ng of purified *ssrA* promoter DNA or a kanamycin resistance gene fragment (kan, negative control) was incubated with increasing concentrations of ArgR protein (0, 0.5, 1, 1.5, and 2 μM) in a 20 μL reaction system containing 1× binding buffer (10 mM Tris–HCl [pH 7.4], 5 mM MgCl_2_, 250 mM KCl, 2.5 mM CaCl_2_, 2.5% [v/v] glycerol, 0.5 mM DTT), in the presence or absence of 10 mM arginine. After incubation at 37 °C for 30 mins, 10 × DNA loading buffer was added to each sample. Complexes were resolved on a 6% native polyacrylamide gel in 0.5 × TBE buffer at 120 V for 90 min. Gels were stained with GelRed for 10 min and visualized using a gel imaging system (Tanon, Shanghai, China).

### 2.15 Statistical analysis

The data are shown as the mean ± standard deviation (SD). Each *in vitro* experiment was performed in duplicate, and at least three independent biological replicates (n ≥ 3) were included. For mouse survival assays, each group included at least ten mice (n ≥ 10); for quantification of bacterial burden (CFU) in liver and spleen, each group included at least three mice (n ≥ 3). All animal experiments were independently performed twice and data from both experiments were pooled for statistical analysis. Statistical analyses were carried out using GraphPad Prism version 9.5.1 (http://www.graphpad.com/, RRID: SCR_002798). To assess the differences between groups, a two-tailed unpaired Student’s t-test, one-way analysis of variance (ANOVA), two-way ANOVA, log-rank (Mantel–Cox) test, or Mann-Whitney U test were employed, as specified in the figure legends. Statistical significance was set at P < 0.05.

## 3 Results

### 3.1 Arginine is accumulated in macrophages in response to *S.* Tm infection

To systematically evaluate the metabolic alterations in macrophages induced by *S*. Tm infection, we conducted untargeted metabolomic profiling of mouse bone marrow-derived macrophages (BMDMs) infected with wild-type *S*. Tm strain 14028s for 8 h, with uninfected BMDMs serving as controls. Metabolites from both groups (*n* = 6 per group) were extracted and analyzed by high-resolution liquid chromatography–mass spectrometry (LC–MS) (**Figure 1A**). A total of 508 and 410 distinct metabolic features were detected in positive (ES+) and negative (ES-) ion modes, respectively. Orthogonal partial least squares discriminant analysis (OPLS-DA) of these features showed a clear separation between the infected and uninfected groups (**Figure 1B**).

**Figure 1:**
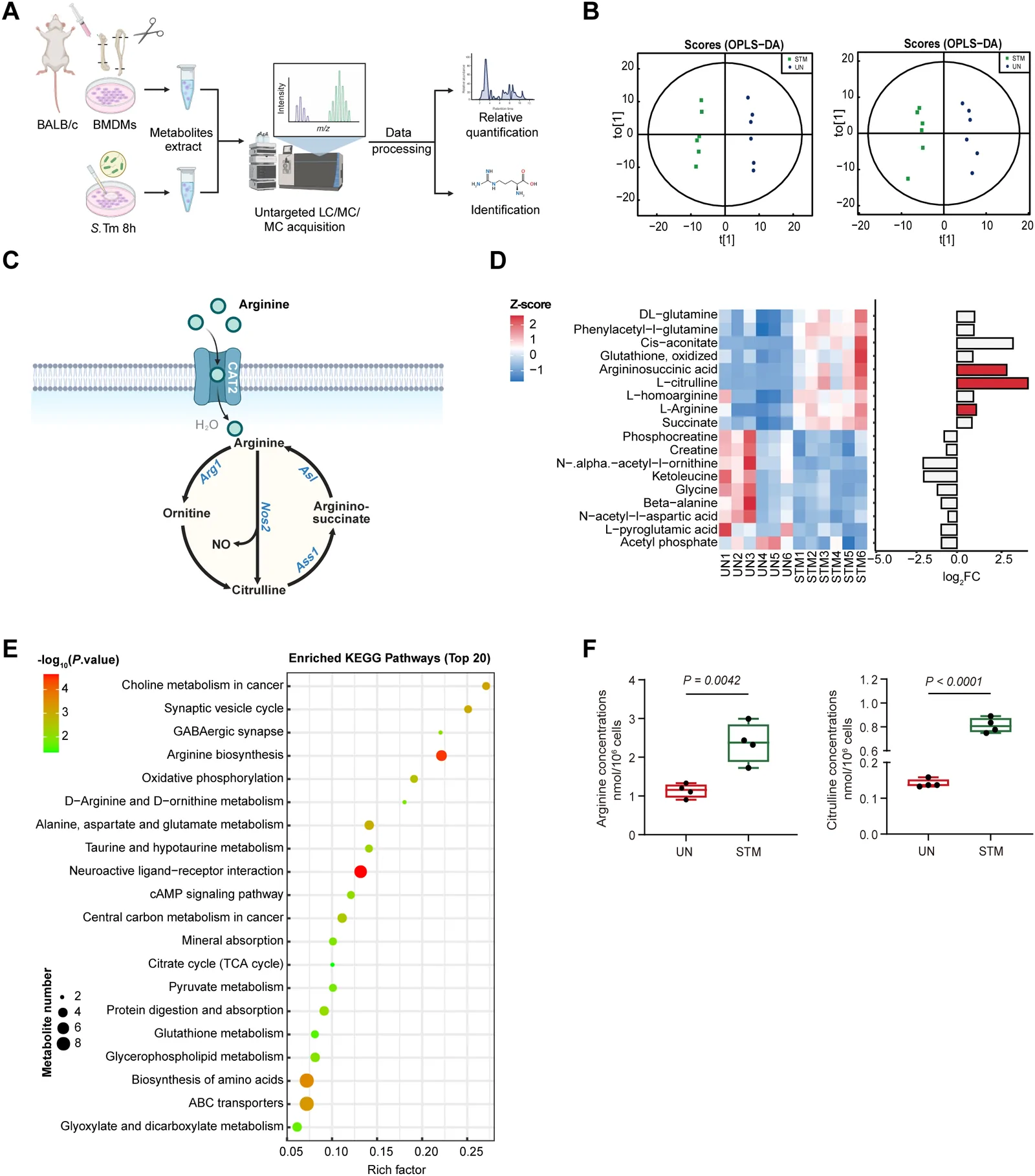
Arginine is accumulated in macrophages in response to *S.* Tm infection. **(A)** Schematic of the experimental workflow for untargeted metabolomics. Created with BioRender.com. **(B)** OPLS-DA score plot generated from UHPLC-Q-TOF/MS data comparing uninfected (UN) and *S*. Tm-infected (STM) bone marrow-derived macrophages (BMDMs) at 8 h post-infection, shown in positive (left) and negative (right) ion modes. A total of 12 mice were randomly divided into 2 groups, with 6 mice in each group. **(C)**, Schematic overview of arginine metabolism in macrophages. CAT2/Slc7a2 represents the eukaryotic arginine transporter. Key enzymes involved include Arg1 (arginase 1), iNOS (inducible nitric oxide synthase), Asl (argininosuccinate lyase), and Ass1 (argininosuccinate synthase 1). Created with BioRender.com. **(D)** Unsupervised hierarchical clustering heatmap and accompanying bar plot illustrating 35 significantly altered amino acids and amino acid-derived organic acids between UN and *S*. Tm-infected BMDMs. Analysis and visualization were performed using OmicStudio tools (https://www.omicstudio.cn/tool). **(E)** KEGG pathway enrichment bubble plot. Bubbles represent the top 20 enriched pathways ranked by *P* value. Bubble size reflects pathway impact in topology analysis, while color intensity corresponds to enrichment significance [−log_10_ (*P* value)]. The rich factor indicates the proportion of differentially abundant metabolites mapped to each pathway. **(F)** Targeted metabolomic quantification of key metabolites within the arginine metabolism pathway in UN and *S*. Tm-infected BMDMs at 8 h post-infection. Data are presented as mean ± SD; *n* = 4 biological replicates per group. Statistical significance was determined by a two-tailed unpaired Student’s *t*-test.

By matching spectral data against molecular weight and structural databases, we annotated the detected features and identified 53 metabolites that were significantly altered in *S.* Tm-infected BMDMs (VIP > 1, *p* < 0.05; **Supplementary Table 3**), among which 21 were upregulated and 32 were downregulated. Of these, 19 metabolites (35.8%) were classified as amino acids or amino acid-derived organic acids. Notably, infection with *S*. Tm resulted in elevated abundances of arginine (2.3-fold) and its metabolic derivatives involved in the iNOS pathway (**Figure 1C**), specifically citrulline (19.2-fold) and argininosuccinate (8.5-fold) (highlighted in **Figure 1D**). Kyoto Encyclopedia of Genes and Genomes (KEGG) pathway enrichment analysis identified arginine metabolism as the most significantly enriched metabolic pathways in infected macrophages (**Figure 1E**). Targeted metabolomic analysis confirmed the accumulation of arginine and citrulline in *S.* Tm-infected BMDMs compared with the control group (*n* = 4 per group; **Figure 1F**). Critically, when compared with the canonical pro-inflammatory M1 macrophage response (IFN-γ + LPS), in which arginine and citrulline have been reported to increase 1.6-fold and 60.1-fold, respectively (Ji et al., 2019), the relative accumulation of citrulline versus arginine in *S.* Tm-infected macrophages (fold-change ratio~8.3) was substantially lower than that in classically activated macrophages (fold-change ratio ~38.0). This difference indicates that iNOS-dependent conversion of arginine to citrulline is restricted during *S.* Tm infection. Collectively, these results demonstrate that *S*. Tm infection significantly influences arginine metabolism in macrophages, driving arginine accumulation while suppressing iNOS-dependent arginine consumption.

### 3.2 Arginine accumulation in infected macrophages correlates with enhanced import and impaired iNOS-dependent consumption

During infection, macrophages upregulate the cationic amino acid transporter 2 (CAT2, encoded by *Slc7a2*) to increase arginine uptake, thereby supporting iNOS-dependent NO production and potentiating the antimicrobial activity of macrophages (Kakuda et al., 1999). Consistent with this, quantitative RT-PCR (qRT-PCR) analysis confirmed increased *Slc7a2* expression in the livers and spleens of *S*. Tm-infected mice compared to uninfected controls (**Figures 2A, B**). Fluorescence *in situ* hybridization (FISH) analysis further demonstrated colocalization of *Slc7a2* mRNA with intracellular *S*. Tm in tissue sections from these organs (**Figure 2C**). Given that *S.* Tm resides predominantly within macrophages in the liver and spleen during systemic infection, this colocalization indicates that *Slc7a2* induction occurs primarily in infected macrophages and is directly associated with *S.* Tm infection. The induction of *Slc7a2* suggests enhanced arginine uptake in *S*. Tm-infected macrophages.

**Figure 2:**
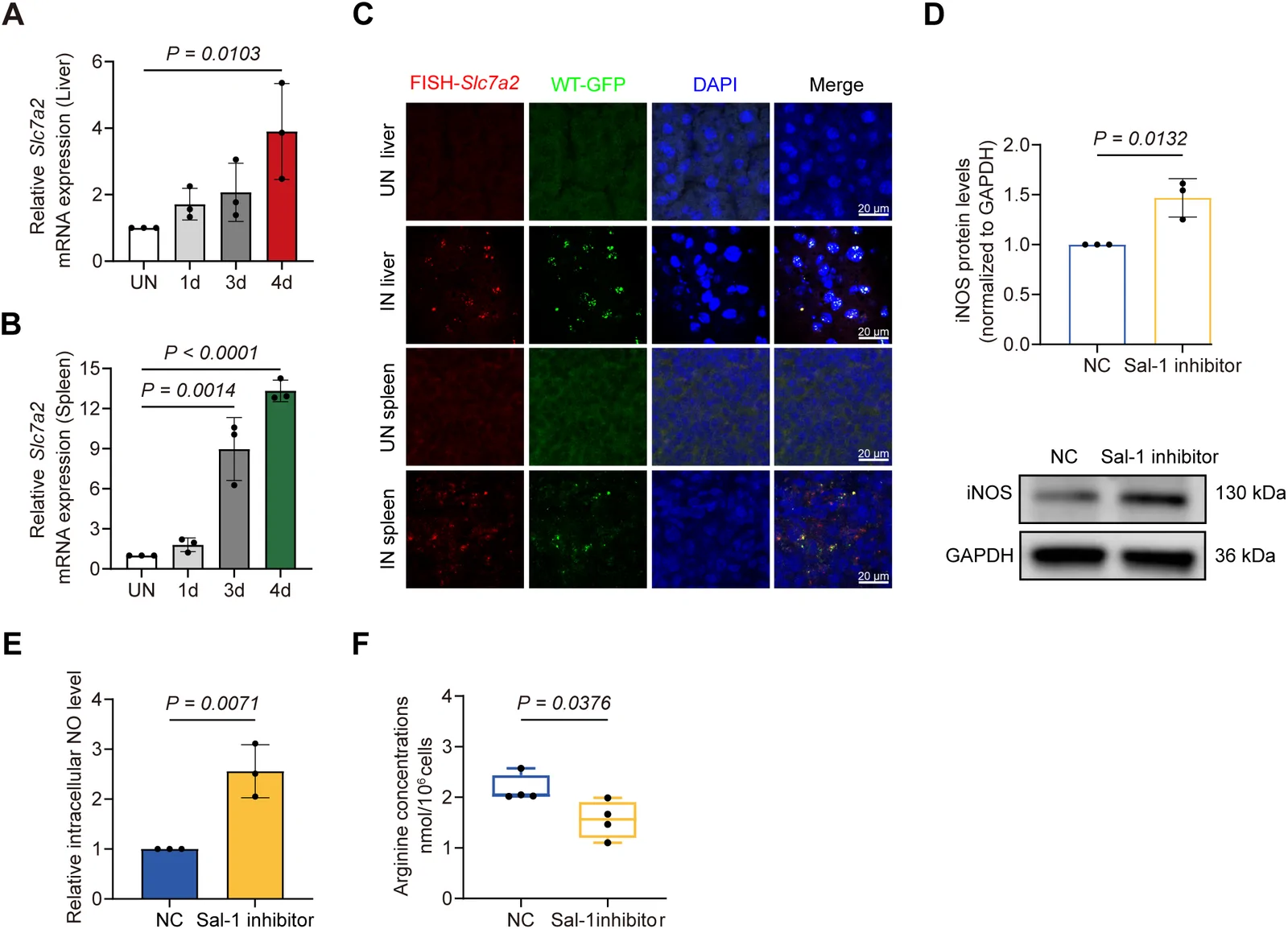
Arginine accumulation in infected macrophages results from *S*. Tm-mediated inhibition of host iNOS expression. **(A, B)**, qRT-PCR analysis of *Slc7a2* mRNA expression in the liver **(A)** and spleen **(B)** of uninfected and *S*. Tm-infected mice at day 1, 3, and 4 post-infection. A total of 12 mice were randomly divided into 4 groups, with 3mice in each group. Data are presented as mean ± SD. **(C)** RNA fluorescence *in situ* hybridization (RNA-FISH) of *Slc7a2* mRNA (red) in uninfected (UN) or *S*. Tm–infected (IN) murine liver (top) and spleen (bottom) at day 4 post-infection. A total of 6 mice were randomly divided into 2 groups, with 3 mice in each group. Nuclei were stained with DAPI (blue); *S.* Tm expressing GFP (green). Scale bars: 20 µm. **(D)** Western blot analysis of iNOS protein levels in BMDMs transfected with Sal-1 inhibitor or negative control miRNA (NC). **(E)** Nitric oxide (NO) production in BMDMs following transfection with Sal-1 inhibitor or NC, as measured by Griess assay. **(F)** Intracellular arginine concentrations in BMDMs transfected with Sal-1 inhibitor or NC, quantified by targeted metabolomics. For **(D–F)**, data are shown as mean ± SD from *n* = 3 **(D, E)** or *n* = 4 **(F)** independent experiments. Statistical significance was determined by one-way ANOVA **(A, B)** or two-tailed unpaired Student’s *t*-test **(D–F)**.

Arginine uptake during infection is primarily dedicated to sustaining NO synthesis for antibacterial defense (Mori and Gotoh, 2000). However, previous studies have shown that *S.* Tm can reduce NO production by suppressing iNOS expression to promote intracellular replication (Gu et al., 2017; Zhao et al., 2017). Notably, the markedly lower citrulline/arginine ratio observed in infected macrophages, when compared with that of classically activated pro-inflammatory macrophages, suggests that arginine accumulation is likely due to impaired iNOS-mediated consumption. To verify whether iNOS suppression results in arginine accumulation in macrophages, we targeted Sal-1, a bacterial small RNA known to repress host iNOS expression, using a specific inhibitor (the reverse complement of Sal-1, specifically neutralizes Sal-1 activity and restores iNOS expression) to alleviate its suppressive effect. Western blot analysis and Griess assay confirmed that inhibition of Sal-1 led to increased iNOS expression and NO production in *S*. Tm-infected BMDMs at 8 h post-infection (**Figures 2D, E**), verifying effective blockade of Sal-1 activity. We then investigated whether Sal-1 inhibition reduces intracellular arginine levels. As expected, metabolomic analysis showed a significant decrease in arginine concentration in inhibitor-treated BMDMs compared to controls (**Figure 2F**). Collectively, these results support a model in which arginine accumulation in *S.* Tm-infected macrophages arises from the combined effect of enhanced arginine import and pathogen-mediated suppression of host iNOS expression.

### 3.3 Arginine accumulation in macrophages is associated with enhanced intracellular *S.* Tm replication

To investigate whether arginine accumulation in macrophages contributes to the intracellular replication of *S*. Tm, we cultured mouse RAW264.7 macrophages in RPMI-1640 medium supplemented with either 1.2 mM or 0.1 mM L-arginine. The 1.2 mM concentration corresponds to the standard arginine content of RPMI-1640 medium and represents an arginine-replete condition (Albina et al., 1989), whereas the 0.1 mM concentration reflects the lower boundary of the reported physiological intracellular range (0.1–1.0 mmol/L) (Martí and Reith, 2021) and models an arginine-limited niche. The reduction in arginine concentration did not affect cell viability, as demonstrated by live cell counting (**Supplementary Figure 1**). At 20 h post-infection, intracellular replication of *S*. Tm was significantly higher (a 1.5-fold increase) in RAW264.7 cells cultured in medium containing 1.2 mM arginine than in those under the 0.1 mM condition (**Figure 3A**), indicating that increased arginine availability enhances intracellular growth of *S.* Tm. To examine whether this effect is mediated by arginine itself or its metabolic products, we inhibited arginine metabolism in RAW264.7 cells using nor-NOHA (an arginase inhibitor) and L-NMMA (an iNOS inhibitor) to block its conversion to ornithine and NO, respectively. RAW264.7 cells were cultured under the two arginine concentrations (1.2 mM and 0.1 mM) in the presence of these inhibitors. Under the metabolic inhibition conditions, *S*. Tm replication remained significantly higher in the 1.2 mM arginine condition than in the 0.1 mM condition (**Figure 3B**), indicating that arginine itself, rather than its downstream metabolites, is associated with enhanced *S.* Tm intracellular replication under these experimental conditions.

**Figure 3:**
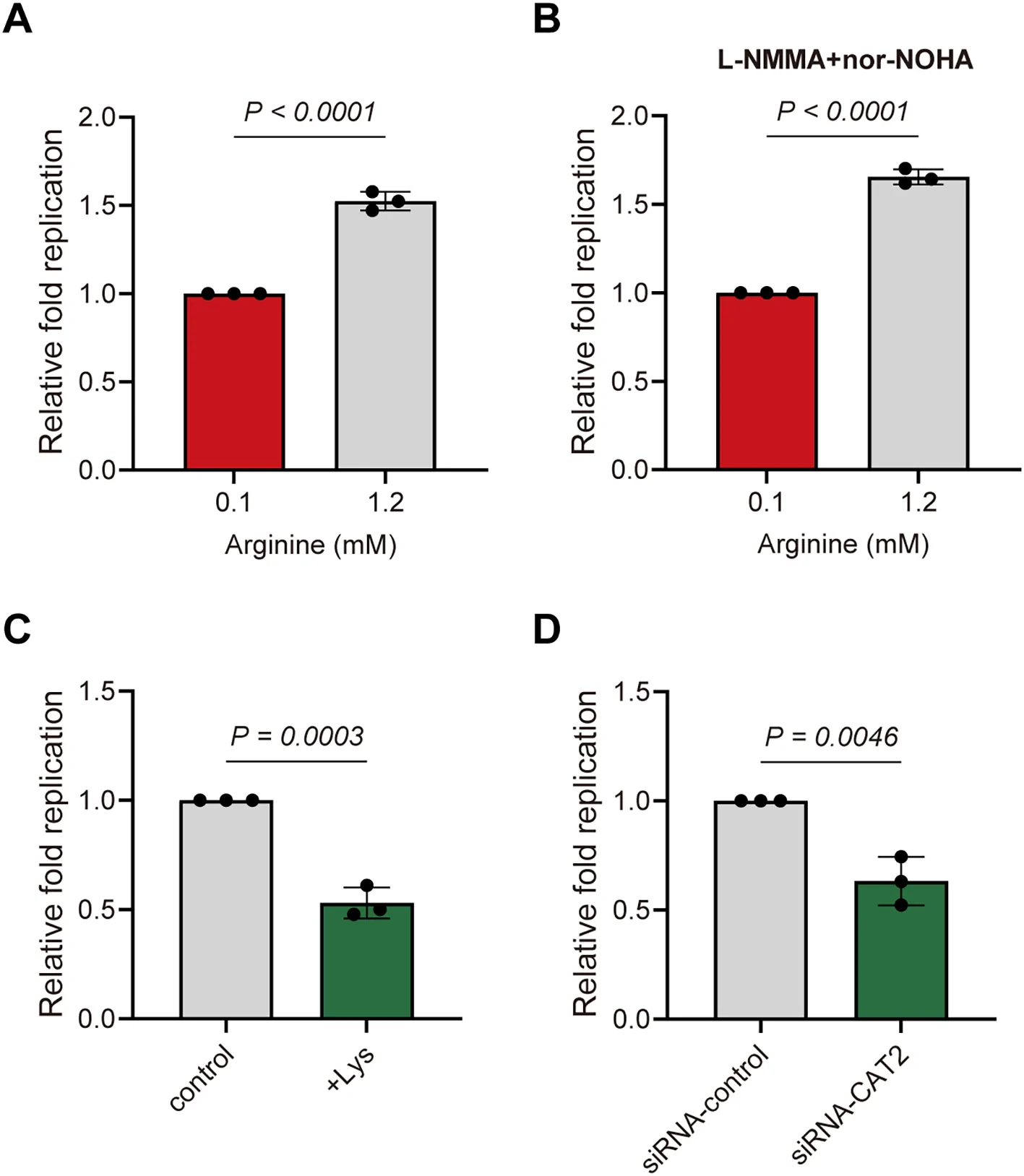
Arginine accumulation in macrophages promotes intracellular replication of *S*. Tm. **(A)** Intracellular replication of *S*. Tm in RAW264.7 cells at 20 h post-infection. Cells were cultured in media supplemented with 0.1 mM or 1.2 mM arginine. **(B)** Intracellular replication of *S*. Tm in RAW264.7 cells at 20 h post-infection. Cells were cultured in arginine-defined media (0.1 mM or 1.2 mM), with co-treatment of 100 μM L-NMMA (iNOS inhibitor) and 100 μM nor-NOHA (arginase inhibitor). **(C)** Intracellular replication of *S*. Tm in RAW264.7 cells at 20 h post-infection following treatment with or without 40 mM L-lysine. **(D)** Intracellular replication of *S.* Tm in RAW264.7 cells at 20 h post-infection after transfection with siRNA targeting CAT2 (siRNA-CAT2) or a non-targeting control (siRNA-control). For all panels **(A–D)**, data are presented as mean ± SD from three independent experiments (*n* = 3). Statistical significance was determined by a two-tailed unpaired Student’s *t*-test.

To further validate the effect of arginine availability in *S*. Tm intracellular replication, we inhibited arginine uptake in RAW264.7 cells using L-lysine, a competitive inhibitor of arginine transport, to deplete the intracellular arginine pool (Devé and Boyd, 1998). Gentamicin protection assays showed that L-lysine treatment resulted in a 2.1-fold decrease in *S*. Tm replication relative to the untreated control (**Figure 3C**). Moreover, siRNA-mediated knockdown of CAT2, a major arginine transporter, decreased *CAT2* mRNA levels by 7.7-fold without affecting macrophage viability (**Supplementary Figure 2**). This knockdown can also decrease macrophage arginine uptake and consequently diminish intracellular arginine pool, leading to a 1.8-fold reduction in bacterial replication in RAW264.7 cells (**Figure 3D**).

Collectively, these results demonstrate that the accumulation of intracellular arginine promotes *S*. Tm replication within macrophages.

### 3.4 SPI-2 T3SS is activated in response to host arginine accumulation to promote *S.* Tm intracellular replication

Since the SPI-2 T3SS is essential for *S*. Tm survival and replication within macrophages, we next tested whether arginine accumulation in macrophages promotes the expression of the SPI-2 genes. To dissociate the effect of arginine from other SCV-associated cues, we performed all assays in acidic N-minimal medium, which recapitulates key physicochemical features of the SCV, acidic pH, low Mg²^+^, and low phosphate, with arginine concentration as the sole variable. Using qRT-PCR and bioluminescent reporter assays, we observed that the mRNA levels of three representative SPI-2 genes (*ssrA*, *ssrB*, and *sifA*) were significantly increased when *S*. Tm was cultured in N-minimal medium supplemented with 1.2 mM arginine (arginine-replete conditions), compared to the no-arginine control (**Figures 4A, B**). Consistent with these findings, Western blot analysis demonstrated that the protein level of the SPI-2 effector SifA was also increased under 1.2 mM arginine supplementation (**Figure 4C**). In contrast, when *S.* Tm was grown in N-minimal medium containing 0.1 mM arginine (arginine-limited conditions), SPI-2 gene expression remained at baseline levels similar to those of the no-arginine control (**Figure 4A**). These results suggest that increased arginine availability promotes SPI-2-dependent gene expression under physiologically relevant *in vitro* conditions.

**Figure 4:**
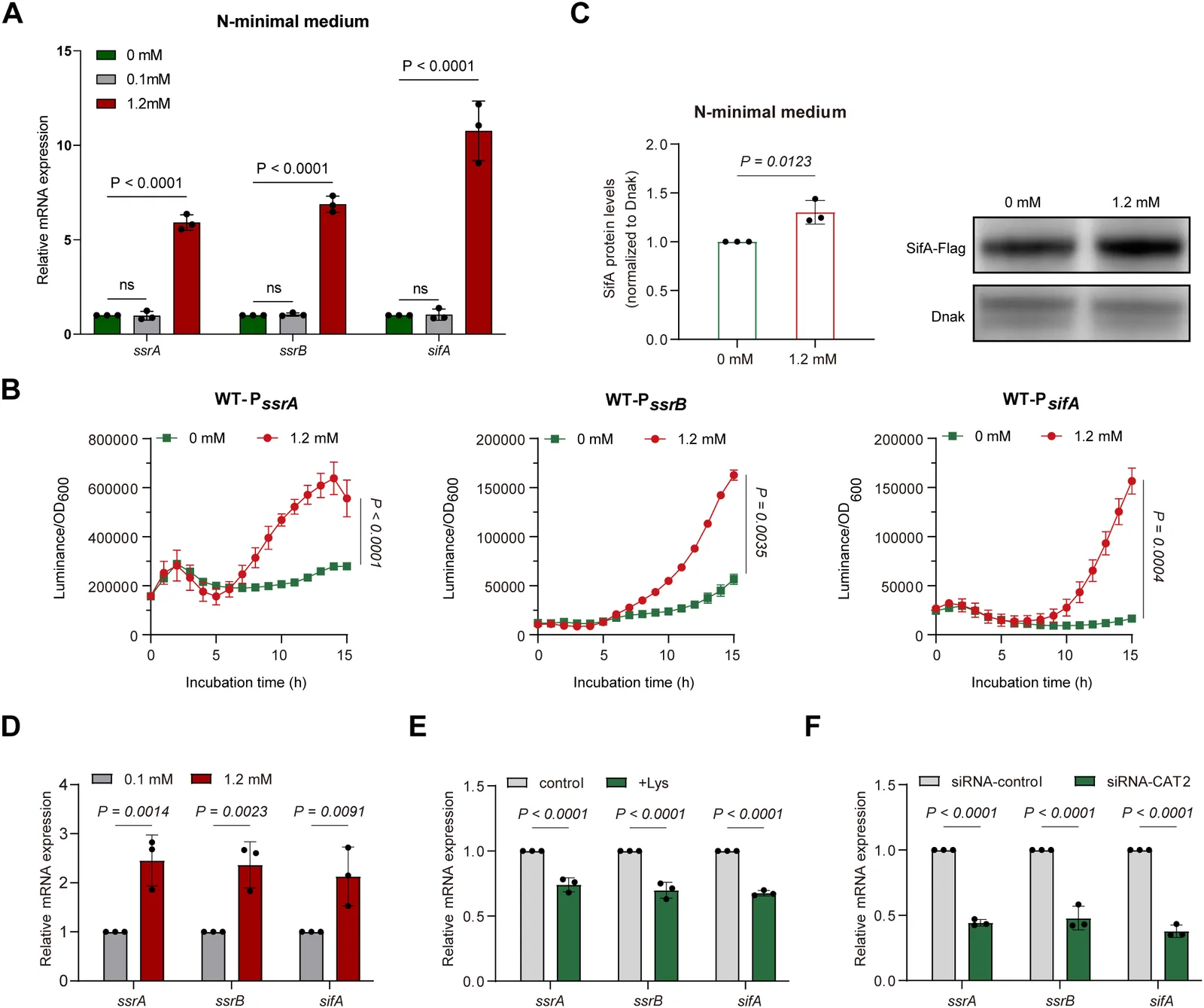
Arginine accumulation promotes *S*. Tm intracellular replication by activating SPI-2 T3SS expression. **(A)** qRT-PCR analysis of mRNA expression for *S*. Tm SPI-2 genes (*ssrA, ssrB, sifA*) cultured in N-minimal medium supplemented with 0.1 mM or 1.2 mM arginine, relative to an arginine-free control. **(B)** Expression of SPI-2 genes (*ssrA, ssrB, sifA*) assessed by bioluminescent reporter assays in *S*. Tm cultured in RPMI 1640 medium with or without 1.2 mM arginine. **(C)** Western blot analysis of SifA protein expression in *S*. Tm cultured in N-minimal medium with or without 1.2 mM arginine. Image is representative of three independent experiments. D–F, qRT-PCR analysis of SPI-2 gene expression (*ssrA, ssrB, sifA*) in *S*. Tm-infected RAW 264.7 cells under the following conditions: **(D)** cultured in medium containing 0.1 mM or 1.2 mM arginine; **(E)** treated with or without 40 mM L-lysine; **(F)** transfected with siRNA targeting CAT2 (siRNA-CAT2) or a non-targeting control (siRNA-control). Data in **(A–F)** are presented as mean ± SD from three independent experiments (*n* = 3). *P* values were determined by two-way ANOVA **(A, D–F)** or two-tailed unpaired Student’s *t*-test **(B, C)**; ns, not significant.

We further validated the role of arginine in promoting SPI-2 expression during macrophage infection. Intracellular *S.* Tm exhibited significantly higher SPI-2 gene expression in RAW264.7 macrophages cultured in RPMI medium containing 1.2 mM arginine than in those containing 0.1 mM arginine (**Figure 4D**). Moreover, reducing intracellular arginine availability, either through competitive inhibition with L-lysine or siRNA-mediated knockdown of the CAT2 transporter, significantly suppressed SPI-2 gene expression (**Figures 4E, F**).

Together, these results demonstrate that host-derived arginine serves as a signal perceived by *S*. Tm to upregulate the SPI-2 T3SS, thereby promoting its intracellular replication.

### 3.5 ArgR activates SPI-2 T3SS expression in response to host arginine

We then investigated how *S.* Tm senses host arginine to activate SPI-2 expression. ArgR (STM3360) is a cytosolic arginine sensor in bacteria. Upon binding arginine, ArgR undergoes allosteric activation and assembles into a hexameric complex that binds ARG box sequences to modulate transcription (Charlier and Bervoets, 2019). Although ArgR is well known to regulate genes involved in arginine biosynthesis, metabolism, and transport, it has also been implicated in controlling T3SS virulence genes in *E. coli* O157 (Menezes-Garcia et al., 2020). We therefore investigated whether ArgR is involved in the arginine-responsive regulation of the SPI-2 T3SS in *S.* Tm.

Using the BPROM program (http://morissardjerome.free.fr/infobiogen/www.softberry.com/berry.html), we identified a putative ArgR-binding sequence within the promoter region of *ssrA*, a key regulator of SPI-2. Electrophoretic mobility shift assays (EMSAs) showed that purified ArgR bound to the promoter region of *ssrA* only in the presence of arginine, whereas a heterologous kanamycin resistance gene (*kan*) showed no detectable binding under any condition tested (**Figure 5A**). These results indicate that ArgR can directly bind to the *ssrA* promoter region in an arginine-dependent manner, supporting the role of ArgR as a direct transcriptional regulator of this locus. The exact nucleotides within this region that constitute the ArgR-binding site remain to be determined.

**Figure 5:**
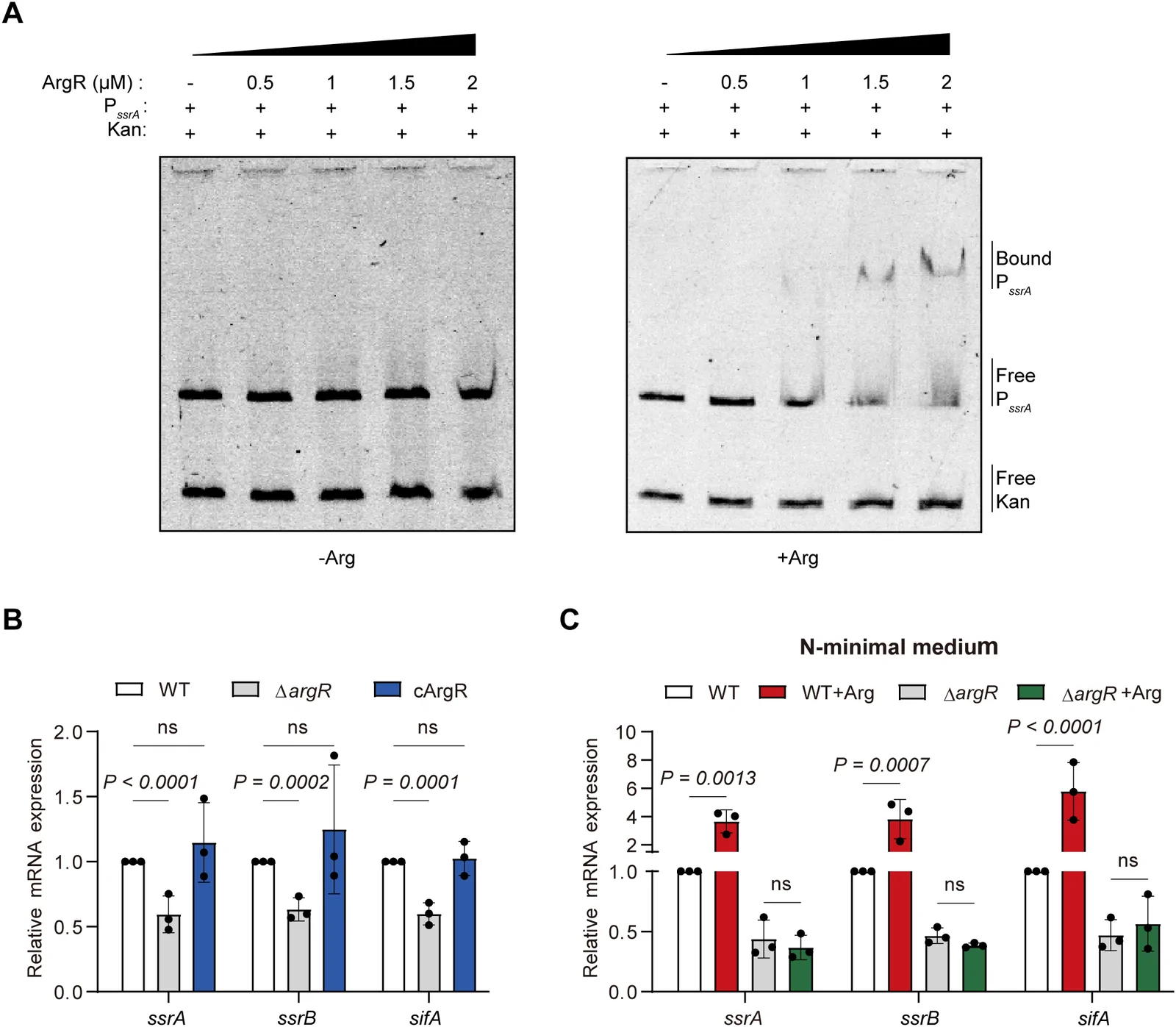
ArgR activates SPI-2 T3SS expression in response to host arginine. **(A)** Electrophoretic mobility shift assay (EMSA) showing binding of purified ArgR-His protein (0–2 μM) to the ssrA promoter region, with a kanamycin resistance gene fragment as negative control. Assays were performed in the presence or absence of arginine. Bound and free DNA are indicated. **(B)** qRT-PCR analysis of SPI-2 gene expression (*ssrA, ssrB, sifA*) in RAW 264.7 cells infected with wild-type (WT) *S*. Tm, *argR* mutant, or *argR*-complemented strains. **(C)** qRT-PCR analysis of SPI-2 gene expression in WT and Δ*argR* mutant *S*. Tm cultured in N-minimal medium with or without 1.2 mM arginine. Data in **(B, C)** are presented as mean ± SD from three independent experiments (*n* = 3). Representative images are shown for **(A)**. *P* values were determined by two-way ANOVA; ns, not significant.

We next constructed an *argR* deletion mutant (Δ*argR*) to assess the role of ArgR in SPI-2 activation. qRT-PCR analysis showed that SPI-2 gene (*ssrA*, *ssrB*, and *sifA*) expression was significantly decreased in the Δ*argR* mutant compared to wild-type strain, during infection of RAW264.7 macrophages at 8 h post-infection (**Figure 5B**). Complementation with the *argR* gene restored SPI-2 transcription to wild-type levels (**Figure 5B**). Moreover, while the addition of 1.2 mM arginine to N-minimal medium significantly induced SPI-2 gene expression in the wild-type strain relative to the no-arginine control, the Δ*argR* mutant exhibited comparable SPI-2 expression levels with or without arginine supplementation (**Figure 5C**). These results demonstrate that ArgR is required for arginine-responsive activation of the SPI-2 genes.

Collectively, these findings demonstrate that ArgR responds to host-derived arginine and activates the expression of SPI-2 T3SS by binding to the *ssrA* promoter region in an arginine-dependent manner.

### 3.6 Activation of SPI-2 by ArgR in response to host arginine is critical for *S*. Tm intracellular replication and systemic infection

To evaluate the significance of ArgR-dependent activation of the SPI-2 T3SS in response to host-derived arginine during *S*. Tm intracellular replication, we infected RAW264.7 macrophages with either wild-type *S*. Tm or the Δ*argR* mutant. Gentamicin protection assays showed a 5.3-fold increase in intracellular CFUs of wild-type *S*. Tm between 2 and 20 h post-infection, whereas the Δ*argR* mutant exhibited significantly attenuated replication, with only a 2.4-fold increase over the same period (**Figure 6A**). The decreased intracellular replication of Δ*argR* mutant was also demonstrated by Immunofluorescence analysis, which showed that RAW264.7 cells contained an average of 5.1 wild-type bacteria per cell at 20 h post-infection, compared to only 2.5 for the Δ*argR* mutant (**Figure 6B**). Deletion of *argR* did not affect bacterial growth in LB, RPMI or N-minimal medium (**Supplementary Figure 3**), ruling out general growth defect as the cause of the observed intracellular replication deficiency. Genetic complementation of the Δ*argR* mutant with *argR* restored intracellular replication to wild-type levels (**Figures 6A, B**), highlighting the importance of ArgR in supporting bacterial replication within macrophages. Increasing the arginine concentration from 0.1 mM to 1.2 mM in the culture medium enhanced intracellular replication of wild-type *S.* Tm in RAW264.7 macrophages but had no effect on the Δ*argR* mutant (**Figure 6C**). Similarly, inhibition of arginine uptake in RAW264.7 cells, via treatment with the competitive inhibitor L-lysine or siRNA-mediated knockdown of CAT2, reduced replication of the wild-type strain, but did not affect the Δ*argR* mutant (**Figures 6D, E**). These results indicate that ArgR is required for sensing host-derived arginine and transducing this signal to drive SPI-2–dependent intracellular replication.

**Figure 6:**
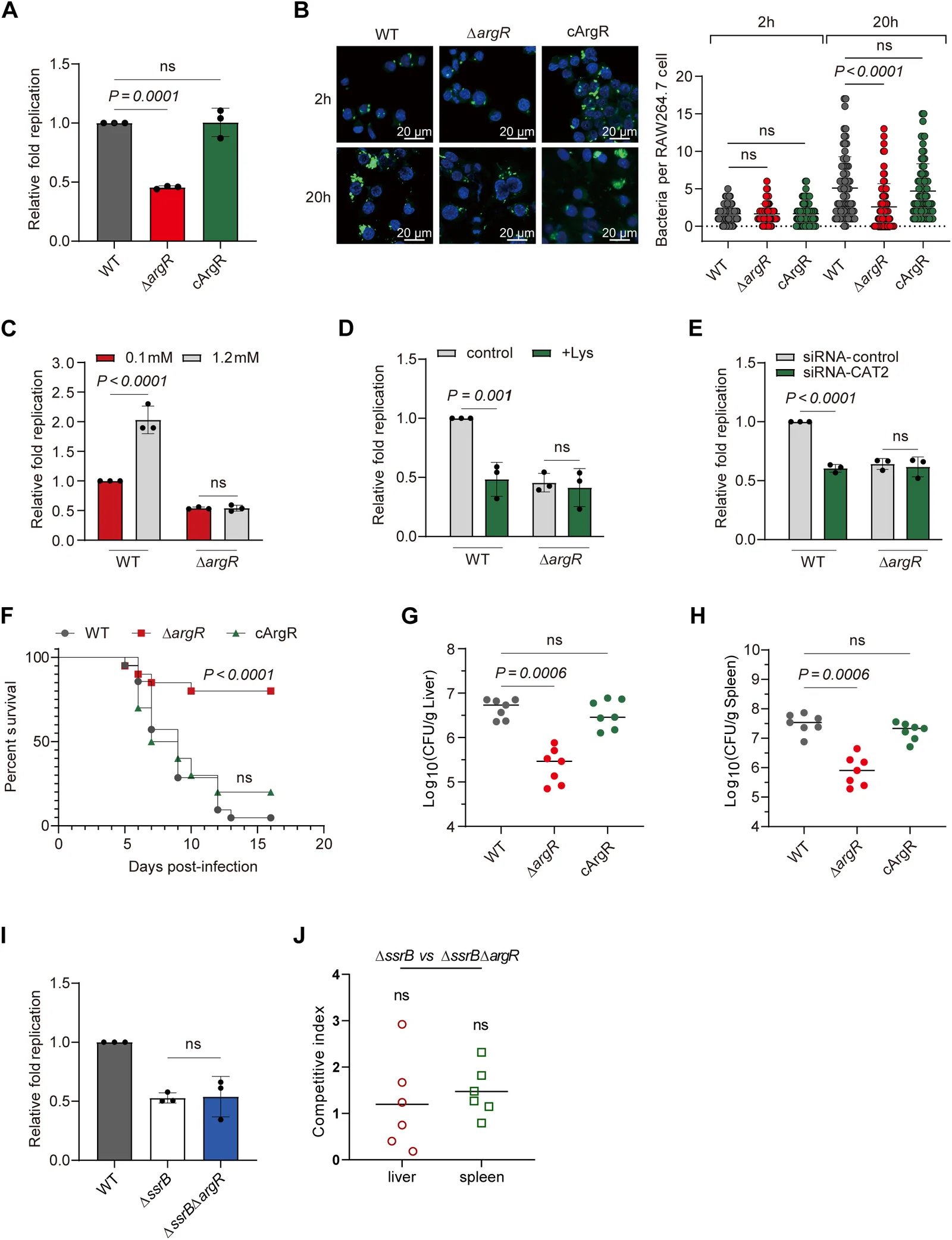
ArgR activation of SPI-2 in response to host arginine is critical for *S*. Tm intracellular replication and systemic infection. **(A)** Intracellular replication of *S*. Tm wild-type (WT), Δ*argR* mutant, and *argR*-complemented strains in RAW264.7 cells at 20 h post-infection. **(B)** Immunofluorescence analysis of infected RAW264.7 cells at 2 and 20 h post-infection. *Salmonella* (green), nuclei (blue). Scale bar, 20 µm (left). Quantification of intracellular bacteria per cell (right) from *n* = 75 cells per group across three independent experiments. **(C–E)** Intracellular replication of *S*. Tm WT and Δ*argR* mutant strains in RAW264.7 cells under the following conditions: **(C)** cultured in medium with 0.1 or 1.2 mM arginine; **(D)** treated with or without 40 mM L-lysine; **(E)** transfected with siRNA targeting CAT2 (siRNA-CAT2) or a non-targeting control (siRNA-control). Infections were carried out for 20 h. **(F)** Survival curves of mice intraperitoneally infected with *S.* Tm WT (*n* = 21), Δ*argR* mutant (*n* = 20), or *argR*-complemented (*n* = 20) strains. A total of 81 mice were randomlydivided into 4 groups. Mice i.p. injected with equivalent dosage of 0.9% NaCl were used as a negative control group (not shown in the figure). **(G, H)** Bacterial burdens in the liver **(G)** and spleen **(H)** of mice infected with the indicated *S*. Tm strains. A total of 28 mice were randomly divided into 4 groups, with 7 mice in each group. Mice i.p. injected with equivalent dosage of 0.9% NaCl were used as a negative control group (not shown in the figure). **(I)** Intracellular replication of *S*. Tm WT, Δ*ssrB* mutant, and Δ*ssrB*Δ*argR* double mutant strains in RAW264.7 cells at 20 hpi. **(J)** Competitive index **(CI)** in liver and spleen of mice co-infected with *S*. Tm Δ*ssrB* mutant versus Δ*ssrB*Δ*argR* double mutant (*n* = 6 mice per group). Data in **(A, C–E, I)** are presented as mean ± SD from three independent experiments (*n* = 3). Representative images are shown in **(B)**. P values were determined by one-way ANOVA **(A, B, I)**, two-way ANOVA **(C–E)**, log-rank (Mantel–Cox) test **(F)**, Mann-Whitney *U* test **(G, H)**, or two-tailed paired Student’s *t*-test on raw CFU values **(J)**. ns, not significant.

We next evaluated the contribution of ArgR activation of SPI-2 to systemic virulence of *S*. Tm *in vivo*. Mice infected intraperitoneally with the Δ*argR* mutant exhibited significantly higher survival rates and reduced bacterial loads in the liver and spleen compared to those infected with the wild-type strain (**Figures 6F–H**). Genetic complementation with *argR* restored systemic virulence to wild-type levels (**Figures 6F–H**), confirming that ArgR-mediated activation of SPI-2 is critical for *S*. Tm full pathogenicity. To investigate whether arginine signaling promotes virulence specifically through SPI-2, we constructed an Δ*ssrB* mutant (impaired in SPI-2 function) and a Δ*ssrB*Δ*argR* double mutant. The double mutant displayed an intracellular replication defect in RAW264.7 cells comparable to that of the Δ*ssrB* single mutant (**Figure 6I**). Competition infection assays showed that the competitive index (CI) of the double mutant versus Δ*ssrB* was approximately 1 in mouse liver and spleen (**Figure 6J**; **Supplementary Figure 4**), indicating that ArgR, and thus the arginine-signaling pathway, promote virulence through SPI-2 activation.

Collectively, these data indicate that ArgR-dependent activation of SPI-2 in response to host arginine is critical for *S*. Tm virulence by enhancing intracellular replication and systemic dissemination of the pathogen.

## 4 Discussion

Macrophages reprogramme metabolic profile during bacterial infection to enhance antibacterial defenses, resulting in substantial alterations in the composition and concentration of intracellular metabolites (Sweet et al., 2025). In response, intracellular pathogens have evolved diverse strategies to exploit or counteract these metabolic changes to their advantage (Jiang et al., 2021; Rosenberg et al., 2021, 2022; Wang et al., 2023; Ma et al., 2025). Although *S*. Tm is known to manipulate macrophage glucose metabolism (Bowden et al., 2009; Jiang et al., 2021), its adaptation to other infection-related metabolic changes remains poorly understood. In this study, we characterized the metabolic alterations in macrophages infected with *S*. Tm. We found that arginine metabolism is markedly affected by *S*. Tm infection, characterized by the accumulation of arginine in host macrophages, which in turn enhances intracellular replication and virulence of *S*. Tm. The mechanism underlying this observation was thoroughly investigated. Based on our findings and existing literature, we propose a model wherein *S*. Tm exploits host arginine to activate virulence programs during infection (**Figure 7**). Specifically, following phagocytosis, arginine uptake is increased in infected macrophages, a response that would normally support iNOS-dependent NO production and antibacterial defense. However, *S*. Tm suppresses the expression of host iNOS to reduce NO production. This combination of increased arginine uptake and reduced iNOS-dependent arginine consumption is associated with the observed arginine accumulation within infected macrophages. The pathogen senses this macrophage-derived arginine via the transcriptional regulator ArgR, which binds to the promoter region of *ssrA* in an arginine-dependent manner to activate the expression of the SPI-2 T3SS, thereby facilitating intracellular bacterial replication and systemic dissemination.

**Figure 7:**
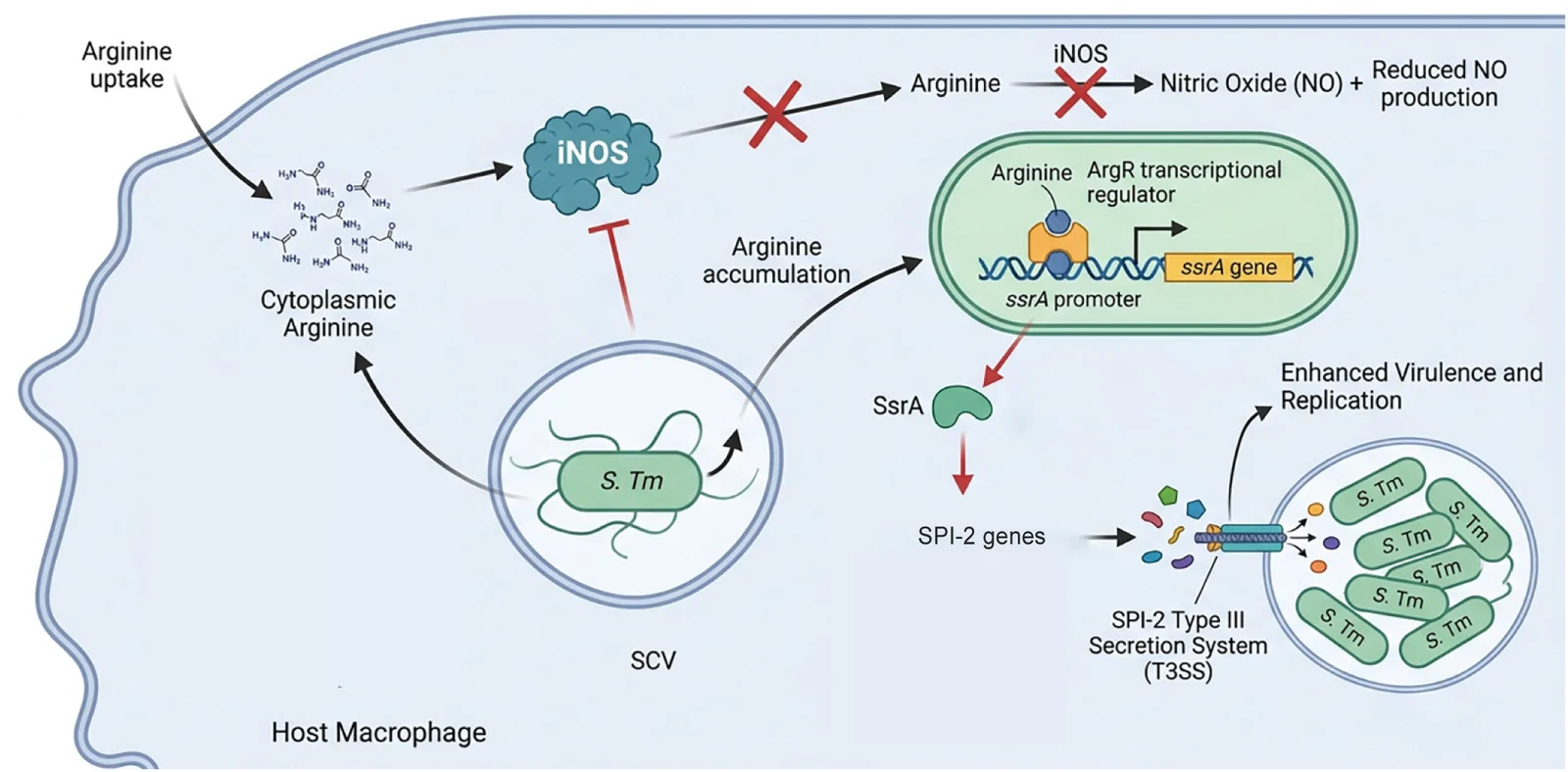
A model for *S*. Tm exploitation of host-derived arginine as a signal to enhance virulence. Upon infection, *S*. Tm induces host arginine uptake, via upregulating cationic amino acid transporter 2 (CAT2), while suppressing inducible nitric oxide synthase (iNOS) expression via Sal-1, leading to intracellular arginine accumulation. The accumulated arginine is sensed by the bacterial transcriptional regulator ArgR, which directly activates the expression of SPI-2 T3SS, thereby promoting bacterial intracellular replication and systemic dissemination.

Arginine plays a crucial role for host immune defense, primarily through its enhanced conversion to NO by iNOS during infection (Mori and Gotoh, 2000; Martí and Reith, 2021). In response to pathogens, macrophages increase arginine uptake to support NO synthesis (Kakuda et al., 1999). Consistent with this, we confirmed that *S*. Tm infection increases arginine import into tissue macrophages. However, *S*. Tm is known to suppress iNOS expression, thereby attenuating NO production and facilitating immune evasion (Gu et al., 2017; Zhao et al., 2017). This dual effect, enhanced uptake coupled with reduced iNOS-dependent consumption, provides a coherent explanation for the observed arginine accumulation. While *S*. Tm can directly assimilate host arginine to support bacterial protein synthesis (Das et al., 2010), our work shows that arginine also serves as a signaling molecule that activates the expression of SPI-2 genes, enhancing bacterial replication within macrophages. Thus, in addition to the established roles of iNOS inhibition-mediated immune evasion and arginine assimilation for biosynthetic purposes, our findings suggest that *S.* Tm senses infection-associated arginine accumulation to activate its intracellular virulence program. Furthermore, a recent study showed that during intestinal colonization, *S*. Tm utilizes arginine generated by the commensal fungus *Candida albicans* to activate SPI-1 T3SS expression and promote epithelial invasion (Jaswal et al., 2025).Collectively, these findings underscore environmental arginine as a pivotal signaling cue that *S*. Tm exploits to facilitate infection across both intestinal and systemic stages.

iNOS-independent mechanisms underlying the observed metabolic profile were also considered. Elevated citrulline and argininosuccinate may derive from urea-cycle activity via the ARG1–OTC and ASS1–ASL axes, or from arginine decarboxylase-driven agmatine and polyamine synthesis, which can secondarily suppress iNOS (Ji et al., 2019). While our data do not exclude such ancillary contributions, three lines of evidence suggest that ARG1/OTC-dependent recycling might not be a major contributor to arginine accumulation. First, the global metabolic signature, arginine accumulation alongside reduced iNOS protein and NO output, and a citrulline-to-arginine ratio markedly lower than in classically activated M1 macrophages with robust iNOS activity, is consistent with impaired iNOS-mediated arginine catabolism as a major mechanism. Second, dominant ARG1/OTC activity would predict pronounced accumulation of ornithine and downstream polyamines, neither of which emerged as significantly altered in our untargeted metabolomic screen. Third, relieving iNOS suppression via inhibition of the bacterial small RNA *Sal-1* significantly reduced intracellular arginine levels, supporting a functional link between iNOS activity and arginine consumption during infection. Collectively, these observations support the interpretation that restricted iNOS-dependent conversion of arginine to citrulline and NO may represent the primary mechanism underlying arginine accumulation, while alternative pathways such as arginine recycling and polyamine metabolism may also contribute to a lesser extent. Future studies employing direct flux measurements will be required to determine the precise quantitative contributions of these pathways.

Intracellular arginine concentrations typically range from 0.10 to 1.00 mM, varying by cell type (Martí and Reith, 2021). Our experiments showed that reducing extracellular arginine or inhibiting its uptake, both resulting in reduced intracellular pools, significantly suppressed SPI-2 gene expression in *S*. Tm and decreased bacterial replication within macrophages. Notably, the expression levels of SPI-2 genes in macrophages cultured under 0.1 mM extracellular arginine were comparable to those under arginine-free conditions, suggesting that SPI-2 activation requires a certain level of arginine availability. Although the exact arginine concentration within infected macrophages has not been precisely quantified, the observed upregulation of SPI-2 genes during infection, along with the critical role of the arginine sensor ArgR in intramacrophage replication and systemic virulence of *S*. Tm, implies that pathogen-induced accumulation raises arginine availability to a level that can support SPI-2 activation. Our findings indicate that *S*. Tm-driven arginine accumulation may be a critical prerequisite for the pathogen to utilize arginine as a signaling molecule.

ArgR functions as a cytosolic arginine sensor in bacteria (Ryan and Mike, 2025). Upon binding arginine, it assembles into a hexameric complex to modulate transcriptional activity (Charlier and Bervoets, 2019). In addition to its canonical roles in regulating arginine biosynthesis, metabolism, and transport, ArgR also regulates virulence genes within the locus of enterocyte effacement (LEE) pathogenicity island in *E. coli* O157, thereby promoting enteric pathogenesis (Menezes-Garcia et al., 2020). The ArgR ortholog in *S.* Tm shares 83% amino acid identity with *E. coli* ArgR, reflecting strong evolutionary conservation between these closely related enteric species. However, its role in virulence gene modulation in *S*. Tm had not been elucidated. In this study, we demonstrate that ArgR binds the *ssrA* promoter region in an arginine-dependent manner and drives its transcriptional activation. These findings support a model in which ArgR links arginine sensing to the SPI-2 regulatory axis in *S.* Tm. These findings expand the regulatory repertoire of ArgR in bacterial pathogenesis and also identify ArgR as a previously unrecognized regulator of the SPI-2 system in *S*. Tm. However, the precise ArgR-binding site within the *ssrA* promoter region has not yet been experimentally defined and will be addressed in future studies.

Taken together, our study has identified arginine as an important metabolic cue in the interaction between *S*. Tm and host macrophages. The arginine-responsive regulator ArgR activates the bacterial virulence program in response to host-derived arginine. Targeting this regulatory axis may offer a promising therapeutic strategy for controlling systemic *Salmonella* infections.

Several limitations of this study should be acknowledged. First, although our data support a model in which arginine accumulation arises largely from enhanced uptake coupled with reduced iNOS-dependent consumption, intracellular arginine flux was not directly measured; thus, the relative contributions of uptake, efflux, and catabolic consumption to the net arginine pool remain to be precisely defined. Second, while EMSA data demonstrate arginine-dependent binding of ArgR to the *ssrA* promoter region, the precise nucleotides constituting the ArgR-binding site have not been experimentally identified. Third, although SPI-2 gene expression was used as a readout for functional activation, direct measurements of SPI-2 T3SS effector secretion were not performed. Addressing these limitations will be important directions for future investigation.

## Data Availability

The source data of this paper are collected in the following database record in Zenodo at http://doi.org/10.5281/zenodo.20808254.
