# Supplementary material for: Salmonella typhimurium exploits macrophage-derived arginine as an intracellular signal for SPI-2 virulence gene activation
Source: Front Cell Infect Microbiol. 2026 Sep 21;16:1877832. doi: 10.3389/fcimb.2026.1877832 (PMC13634974; doi:10.3389/fcimb.2026.1877832)
Supplement: Supplementary file 1 [file SupplementaryFile1.docx]

Supplementary Material

# 1 Supplementary Figures and Tables

## 1.1 Supplementary Figures

**Supplementary Figure 1. Viability of RAW264.7 cells cultured in medium with different arginine concentrations.** RAW264.7 cells were cultured in RPMI medium containing 0.1 mM or 1.2 mM arginine for 20 h, and viable cell numbers were quantified. Data are presented as mean ± SD from three independent experiments (*n* = 3). *P* values were determined by two-tailed unpaired Student's *t*-test; ns, not significant.

**Supplementary Figure 2. qRT-PCR analysis of CAT2 mRNA expression in** RAW 264.7 cells transfected with control siRNA or CAT2‑specific siRNA. Data are presented as mean ± SD from three independent experiments (n = 3). *P* values were determined by two-tailed unpaired Student's *t*-test.

**Supplementary Figure 3. Growth curves of *S.* Tm wild-type (WT), Δ*argR* mutant, and *argR*-complemented strains in**  in three different media: (A) LB, (B) RPMI 1640, and (C) N‑minimal medium. Data are presented as mean ± SD from three independent experiments (n = 3).

**Supplementary Figure 4.**  Bacterial burdens of the Δ*ssrB* and Δ*ssrB*Δ*argR* mutants in the liver (A) and spleen (B) of mice co‑infected with a 1:1 mixed inoculum of the two strains. Data are from six mice (n = 6). Statistical significance was determined using the Mann–Whitney U test; ns, not significant.

## 1.2 Supplementary Tables

Supplementary Table 1. Strains and plasmids used in this study.

| Strains | Genotype or description | Source |
| --- | --- | --- |
| WT *S.* Tm | Wild-type *S.* Typhimurium ATCC 14028s | ATCC* |
| DH5α | *E. coli* DH5α strain | Lab collection |
| BL21(DE3) | *E. coli* BL21(DE3) strain | Lab collection |
| Δ*argR* | WT *S.* Tm strain *argR*::Cm; Cm^R^ | This study |
| Δ*ssrB* | WT *S.* Tm strain *ssrB*::Km; Km^R^ | This study |
| Δ*ssrB*Δ*argR* | WT *S.* Tm strain *argR*::Cm and *ssrB*::Km; Cm^R^, Km^R^ | This study |
| cArgR | Δ*argR* strain *phoN*::*argR*; Cm^R^, Km^R^ | This study |
| BL21-*argR* | BL21 containing pET28a-*argR* | This study |
| WT+*ssrA-*lux | WT *S.* Tm containing plasmid *ssrA-*lux; Km^R^ | This study |
| WT+*ssrB-*lux | WT *S.* Tm containing plasmid *ssrB-*lux; Km^R^ | This study |
| WT+*sifA-*lux | WT *S.* Tm containing plasmid *sifA-*lux; Km^R^ | This study |
| WT-GFP | WT *S.* Tm containing plasmid pCMV-C-EGFP; Ap^R^ | Lab collection |
| *sifA*-FLAG | WT *S.* Tm *sifA* gene tagged with 3×FLAG tag; Cm^R^ | Lab collection |
| Plasmids | | |
| pKD46 | λ-Red recombinase system under an arabinose-inducible promoter; Ap^R^ | Lab collection |
| pKD3 | template plasmid containing a chloramphenicol resistance cassette and the flipase recognition sites; Cm^R^ | Lab collection |
| pKD4 | template plasmid containing a kanamycin resistance cassette and the flipase recognition sites; Km^R^ | Lab collection |
| pCP20 | temperature-sensitive replicon expressing the FLP gene to abolish the antibiotic resistance of the mutant strains; Ap^R^ | Lab collection |
| pET28a | T7 expression vector, Km^R^ | Lab collection |
| pMS402 | containing a promoterless *luxCDABE* reporter gene cluster; Km^R^ | Lab collection |
| pCMV-C-EGFP | expressing EGFP (Enhanced Green fluorescent protein); Ap^R^ | Lab collection |
| pET28a-*argR* | pET28a carrying the *argR* gene; Km^R^ | This study |
| *ssrA-*lux | pMS402 carrying the *ssrA* gene promoter region; Km^R^ | This study |
| *ssrB-*lux | pMS402 carrying the *ssrB* gene promoter region; Km^R^ | This study |
| *sifA-*lux | pMS402 carrying the *sifA* gene promoter region; Km^R^ | This study |

*ATCC, American Type Culture Collection.

Supplementary Table 2. Primers used in this study (5'–3')

| Primers for gene mutation | | |
| --- | --- | --- |
| *argR* | F | ataaaaattcatctgtatgcacaataatgttgtttctaccgccatattacgggtgacttGTGTAGGCTGGAGCTGCTTCG |
|  | R | agcggcgaggggagaggacaaaaaggtgcctccgccgtacgacgacggaggaagtgggaCATATGAATATCCTCCTTAG |
| *ssrB* | F | tgcgtggcgtaaggctcatcaaaatatgaccaatgcttaataccatcggacgcccctggGTGTAGGCTGGAGCTGCTTCG |
|  | R | tgatcttcaaaaactacaccattacttaatattatcttaattttcgcgagggcagcaaaCATATGAATATCCTCCTTAG |
| *sifA*-FLAG | P1 | CGCTACACGTTCGCTCAGAACAACAAAGCGGCTGTTTATGTTGTTTTTTAGACTACAAAGACCATGACGGTGATT |
|  | P2 | CGAAGCAGCTCCAGCCTACACTTACTATTTATCGTCGTCATCTTTGTAGTC |
|  | P3 | GACTACAAAGATGACGACGATAAATAGTAAGTGTAGGCTGGAGCTGCTTCG |
|  | P4 | AGTACGTGAGTAAACCCTGAACGTGACGTCTGAGAAAGCGTCGTCTGATTCATATGAATATCCTCCTTAGTTCCTATTC |
| Primers for identification of the mutants | | |
| *argR* | F | cggcggcgaccttaattca |
|  | R | ataggttctgcggcgaaag |
| *ssrB* | F | gatgtccgccatcagtacgc |
|  | R | cgctgctcagcgctactg |
| *sifA*-FLAG | F | AGCGGATAAGACGTTTCAGGC |
|  | R | AGTACGTGAGTAAACCCTGAACGTGACGTCTGAGAAAGCGTCGTCTGATTCATATGAATATCCTCCTTAGTTCCTATTC |
| Primers for complement construction | | |
| *argR* | P1 | ATGAAAAGTCGTTATTTAGTATTTTTTCTACCACTGATCGACCTTAATTCACAAATTC |
|  | P2 | CGAAGCAGCTCCAGCCTACACTACGACGACGGAGGAAGTG |
|  | P3 | CACTTCCTCCGTCGTCGTAGTGTAGGCTGGAGCTGCTTCG |
|  | P4 | TCAGTAATTAAGTTTGGGGTGATCTTCTTTACTCAATACATATGAATATCCTCCTTAG |
| Primers for gene cloning | | |
| *ssrA*-lux | F | CGGGATCCGATGTTTGCAGCGTATTCTTG |
|  | R | CCGCTCGAGTTCGCTCACAACCACATCC |
| *ssrB*-lux | F | CGGGATCCccattagcacctgcggctaaag |
|  | R | CCGCTCGAGggaggaactaaccgacttacgc |
| *sifA*-lux | F | CGGGATCCgcgttcggcaaacaacatctc |
|  | R | CCGCTCGAGcctgtagcgacgctatcacag |
| Primers for protein purification | | |
| *argR* | F | CGggatcCatgcgaagctcggctaaac |
|  | R | CGgAATTCctaaagctcttgttcaaaaagttccag |
| Primers for EMSA | | |
| P*ssrA* | F | CCTTGTCACAGGCGATTCTA |
|  | R | CCATTGATATATAAGCTGCG |
| Kan | F | gggcaggatctcctgtcatc |
|  | R | cctgatgctcttcgtccagat |
| Primers for qRT-PCR | | |
| *Gapdh* | F | TGTAGACCATGTAGTTGAGGTCA |
|  | R | AGGTCGGTGTGAACGGATTTG |
| *Slc7a2* | F | GTGAAGAGGTTCGGAATCCACA |
|  | R | CGTTAAAGCTGCAGAAACCCC |
| *16S rRNA* | F | GAAAGCGTGGGGAGCAAAC |
|  | R | ACATGCTCCACCGCTTGTG |
| *ssrA* | F | CTGGACCTCTTGCTGGCTGAT |
|  | R | TGGCGTAAGTCGGTTAGTTCCT |
| *ssrB* | F | AAGTTCTGTTAGCGGCATTGC |
|  | R | AGCAGTTGATGATTGGTCGTGT |
| *sifA* | F | ATGATGCCACCATTATTCTTCG |
|  | R | CGTCATTTGTGGATGCGATT |

**Supplementary Table 3.** 53 metabolites that showed significant changes between *Salmonella* Typhimurium (*S*. Tm)-infected and uninfected mouse bone marrow-derived macrophages (BMDMs).

| Ion mode | Name | VIP | Fold change | *P*-value | m/z | rt (s) | HMDB | KEGG | SuperClass |
| --- | --- | --- | --- | --- | --- | --- | --- | --- | --- |
| ES+ | 2-aminoethylphosphonic acid | 1.68 | 0.51 | 0.0002 | 126.05 | 518.19 | HMDB0011747 | C03557 | Organic acids and derivatives |
| ES+ | L-citrulline | 4.66 | 19.21 | 0.0006 | 198.08 | 720.21 | HMDB0000904 | C00327 | Organic acids and derivatives |
| ES+ | Arginine | 18.33 | 2.29 | 0.0007 | 175.12 | 964.50 | HMDB0000517 | C00062 | Organic acids and derivatives |
| ES+ | Argininosuccinic acid | 1.82 | 8.45 | 0.0010 | 291.13 | 864.33 | HMDB0000052 | C03406 | Organic acids and derivatives |
| ES+ | DL-glutamine | 6.04 | 2.12 | 0.0036 | 147.08 | 674.83 |  | C00303 | Organic acids and derivatives |
| ES+ | Creatine | 3.98 | 0.64 | 0.0054 | 132.08 | 812.47 | HMDB0000064 | C00300 | Organic acids and derivatives |
| ES+ | N-.alpha.-acetyl-l-ornithine | 1.49 | 0.24 | 0.0069 | 115.09 | 355.12 | HMDB0003357 | C00437 | Organic acids and derivatives |
| ES+ | L-homoarginine | 1.04 | 2.02 | 0.0106 | 189.13 | 972.71 | HMDB0000670 | C01924 | Organic acids and derivatives |
| ES+ | Glutathione, oxidized | 1.68 | 1.97 | 0.0136 | 613.16 | 928.99 | HMDB0003337 | C00127 | Organic acids and derivatives |
| ES+ | Phosphocreatine | 9.05 | 0.58 | 0.0156 | 212.04 | 812.28 | HMDB0001511 | C02305 | Organic acids and derivatives |
| ES+ | N-acetyl-l-aspartic acid | 2.22 | 0.69 | 0.0173 | 176.04 | 621.39 | HMDB0000812 | C01042 | Organic acids and derivatives |
| ES+ | Beta-alanine | 3.21 | 0.51 | 0.0461 | 90.06 | 655.28 | HMDB0000056 | C00099 | Organic acids and derivatives |
| ES- | Succinate | 2.67 | 1.90 | < 0.0001 | 117.02 | 704.50 | HMDB0000254 | C00042 | Organic acids and derivatives |
| ES- | Phenylacetyl-l-glutamine | 2.98 | 2.07 | 0.0002 | 145.06 | 678.62 | HMDB0006344 | C04148 | Organic acids and derivatives |
| ES- | Cis-aconitate | 5.28 | 10.11 | 0.0005 | 129.02 | 667.22 | HMDB0000072 | C00417 | Organic acids and derivatives |
| ES- | Ketoleucine | 4.53 | 0.24 | 0.0145 | 129.05 | 75.95 | HMDB0000695 | C00233 | Organic acids and derivatives |
| ES- | Guanidinoethyl sulfonate | 1.12 | 0.50 | 0.0286 | 166.02 | 361.13 | HMDB0003584 | C01959 | Organic acids and derivatives |
| ES- | Glycine | 1.39 | 0.43 | 0.0433 | 74.02 | 655.93 | HMDB0000123 | C00037 | Organic acids and derivatives |
| ES- | L-pyroglutamic acid | 3.54 | 0.51 | 0.0452 | 128.03 | 538.21 | HMDB0000267 | C01879 | Organic acids and derivatives |
| ES+ | 1-oleoyl-sn-glycero-3-  phosphocholine | 3.06 | 2.98 | < 0.0001 | 522.36 | 337.00 | HMDB0002815 |  | Lipids and lipid-like molecules |
| ES+ | 1-heptadecanoyl-sn-glycero-3-phosphocholine | 1.21 | 1.91 | 0.0001 | 510.36 | 338.87 | HMDB0012108 |  | Lipids and lipid-like molecules |
| ES+ | Lpc 18:1 | 2.33 | 2.97 | 0.0001 | 522.36 | 305.74 |  |  | Lipids and lipid-like molecules |
| ES+ | Glycerophosphocholine | 27.47 | 2.49 | 0.0002 | 258.11 | 703.61 | HMDB0000086 | C00670 | Lipids and lipid-like molecules |
| ES+ | L-palmitoylcarnitine | 2.51 | 0.64 | 0.0019 | 400.34 | 304.73 | HMDB0000222 | C02990 | Lipids and lipid-like molecules |
| ES+ | 1-palmitoyl-sn-glycero-3-  phosphocholine | 4.00 | 1.65 | 0.0095 | 496.34 | 341.50 | HMDB0010382 |  | Lipids and lipid-like molecules |
| ES+ | 5-aminovaleric acid betaine | 2.15 | 0.53 | 0.0249 | 160.13 | 701.68 |  |  | Lipids and lipid-like molecules |
| ES+ | 1-Stearoyl-sn-glycerol 3-phosphocholine(LPC(18:0)) | 3.50 | 1.42 | 0.0251 | 524.37 | 335.40 | HMDB0010384 | C04230 | Lipids and lipid-like molecules |
| ES+ | Propionylcarnitine | 4.14 | 0.62 | 0.0354 | 218.14 | 497.81 | HMDB0000824 |  | Lipids and lipid-like molecules |
| ES+ | 1,2-dioleoyl-sn-glycerol | 1.05 | 0.74 | 0.0427 | 339.29 | 53.08 | HMDB0007218 |  | Lipids and lipid-like molecules |
| ES- | 13-hydroxy-9z,11e-  octadecadienoic acid | 2.90 | 34.56 | < 0.0001 | 299.26 | 152.09 | HMDB0112194 |  | Lipids and lipid-like molecules |
| ES- | Glycerophosphate(2) | 1.35 | 2.20 | 0.0003 | 171.01 | 709.76 | HMDB0000126 | C00093 | Lipids and lipid-like molecules |
| ES- | Linoleic acid | 2.05 | 0.62 | 0.0206 | 279.23 | 57.41 | HMDB0000673 | C01595 | Lipids and lipid-like molecules |
| ES- | Nonanoic acid | 1.29 | 0.69 | 0.0430 | 157.12 | 93.16 | HMDB0000847 | C01601 | Lipids and lipid-like molecules |
| ES- | Pg 44:12 | 1.07 | 0.69 | 0.0500 | 865.50 | 50.20 |  |  | Lipids and lipid-like molecules |
| ES+ | Arachidonoyl ethanolamide | 11.87 | 0.17 | 0.0001 | 348.31 | 165.92 | HMDB0004080 | C11695 | Organic nitrogen compounds |
| ES+ | Heptadecasphinganine | 7.06 | 1.39 | 0.0001 | 288.29 | 56.43 |  |  | Organic nitrogen compounds |
| ES+ | Sphinganine | 1.09 | 0.21 | 0.0002 | 302.31 | 276.58 | HMDB0000269 | C00836 | Organic nitrogen compounds |
| ES+ | Phytosphingosine | 3.03 | 0.54 | 0.0032 | 318.30 | 60.59 | HMDB0004610 | C12144 | Organic nitrogen compounds |
| ES+ | Docosahexaenoyl ethanolamide | 1.44 | 1.60 | 0.0152 | 372.29 | 155.48 | HMDB0013658 |  | Organic nitrogen compounds |
| ES+ | Acetylcholine | 3.56 | 0.58 | 0.0293 | 146.12 | 686.23 | HMDB0000895 | C01996 | Organic nitrogen compounds |
| ES+ | Pterine | 2.80 | 1.97 | 0.0062 | 164.06 | 323.03 | HMDB0000802 | C00715 | Organoheterocyclic compounds |
| ES+ | Adenine | 1.79 | 0.63 | 0.0104 | 136.05 | 621.64 | HMDB0000034 | C00147 | Organoheterocyclic compounds |
| ES+ | 3-hydroxyanthranilic acid | 2.11 | 0.60 | 0.0082 | 154.06 | 621.64 | HMDB0001476 | C00632 | Benzenoids |
| ES- | Dopamine | 1.31 | 0.56 | 0.0262 | 152.00 | 361.20 | HMDB0000073 | C03758 | Benzenoids |
| ES+ | N-acetyl-d-glucosamine | 1.01 | 0.55 | 0.0237 | 204.09 | 790.23 | HMDB0000215 | C00140 | Organic oxygen compounds |
| ES+ | 4-hydroxyhexenal | 1.47 | 0.55 | 0.0263 | 97.04 | 122.24 |  |  | Organic oxygen compounds |
| ES+ | Udp-n-acetylglucosamine | 1.05 | 0.59 | 0.0169 | 608.09 | 790.23 | HMDB0000290 | C00043 | Nucleosides, nucleotides, and analogues |
| ES+ | S-methyl-5'-thioadenosine | 7.88 | 1.72 | 0.0269 | 298.10 | 168.17 | HMDB0001173 | C00170 | Nucleosides, nucleotides, and analogues |
| ES- | Uridine 5'-diphosphate | 1.05 | 0.50 | 0.0006 | 402.99 | 861.99 | HMDB0000295 | C00015 | Nucleosides, nucleotides, and analogues |
| ES- | Phosphoric acid | 3.63 | 0.74 | 0.0026 | 96.97 | 849.60 | HMDB0000973 | C00009 | Homogeneous non-metal compounds |
| ES+ | 1-(1z-octadecenyl)-2-  (4z,7z,10z,13z,16z,19z-  docosahexaenoyl)-sn-glycero-3-phosphoethanolamine | 2.24 | 0.61 | 0.0001 | 385.27 | 51.12 |  |  |  |
| ES+ | .beta.-nicotinamide adenine dinucleotide(NAD) | 1.72 | 0.60 | 0.0014 | 664.12 | 800.05 | HMDB0000902 | C00003 |  |
| ES+ | Adenosine 3',5'-diphosphate (PAP) | 1.39 | 0.65 | 0.0036 | 445.06 | 812.28 |  |  |  |
